# Lebanese pharmacists’ confidence and self-perceptions of computer literacy: scale validation and correlates

**DOI:** 10.1186/s40545-020-00246-y

**Published:** 2020-08-24

**Authors:** Souheil Hallit, Samah Tawil, Hala Sacre, Clara Rahme, Aline Hajj, Pascale Salameh

**Affiliations:** 1 Faculty of Medicine and Medical Sciences, Holy Spirit University of Kaslik (USEK), Jounieh, Lebanon; 2 INSPECT-LB: Institut National de Santé Publique, Epidémiologie Clinique et Toxicologie, Beirut, Lebanon; 3 Drug Information Center, Order of Pharmacists in Lebanon, Beirut, Lebanon; 4 Faculty of Science, Lebanese University, Fanar, Lebanon; 5 Faculty of Pharmacy, Saint-Joseph University, Beirut, Lebanon; 6 Laboratoire de Pharmacologie, Pharmacie Clinique et Contrôle de Qualité des Médicaments, Saint-Joseph University, Beirut, Lebanon; 7 Faculty of Pharmacy, Lebanese University, Beirut, Lebanon; 8 Faculty of Medicine, Lebanese University, Beirut, Lebanon

**Keywords:** Computer, Literacy, Continuing education, Pharmacist, Lebanon

## Abstract

**Background:**

Most pharmacists agree that continuing education (CE) programs positively affect their practice and increase their knowledge, with computer-based courses being their preferred type of CE (60.6%). The route to using medical e-services and e-learning is not straightforward. High setup costs and time commitments to sustain quality are issues brought up in this respect. Therefore, the objectives of this study were to validate the general confidence with computer use (GCWCU) scale and assess computer literacy and its associated factors among Lebanese pharmacists.

**Methods:**

This cross-sectional study was conducted, using a random sample of Lebanese pharmacists from all districts of Lebanon. The general confidence with computer use (GCWCU) scale was used to assess computer literacy.

**Results:**

This study enrolled 628 (83.73%) pharmacists. The GCWCU items converged over two factors (Cronbach’s alpha = 0.716). A higher GCWCU was associated with the ease of access to the Order of Pharmacists of Lebanon e-library (beta = 2.58), owning a tablet (beta = 2.80), being connected for 4 h daily (beta = 2.71), the ease of access to the learning management system (beta = 2.39), holding a PhD (beta = 4.28) or a PharmD (beta = 1.16), and working in hospitals (beta = 2.60).

**Conclusion:**

This study showed that the GCWCU is adequate to assess computer literacy in Lebanese pharmacists and identified factors affecting and/or associated with computer literacy. It presented insights into essential computer skills and abilities of Lebanese pharmacists and identified factors associated with their general confidence with computer use in their practice. These findings would help decision-makers and CE providers design learning materials for pharmacists to improve their computer literacy for better practice and patient care.

## Background

Pharmacy is an intensive counseling and information profession that has been steadily shifting towards patient monitoring and communication with other healthcare professionals. Nowadays, with data integration and use, information, knowledge, and technology, computer use is becoming an essential component of the pharmacy profession [1–3]. Therefore, pharmacists are required to manage healthcare information, operate a computer, and keep up with advances in information technology; this would allow them to access, recover, analyze, and store large amounts of information related to all patients [4]. Computer skills, along with strong clinical competencies, are necessary to provide optimal patient-related services [5].

In developing countries, such as Nigeria and India, a solid overall knowledge of computers has been demonstrated among healthcare professionals, mainly physicians and nurses, but less among pharmacists [6–8]. On the other hand, studies on the use of the Internet for clinical practice purposes are abundant, particularly in developed countries, but also in Brazil, a developing country where pharmacists make frequent use of the Web and digital instruments to assist them in their clinical practice [9]. In developed countries, the majority of pharmacists use computers extensively, whether at work or home or both, with rates differing by country: TX, USA (98%) [10]; Australia (73.3%) [11]; and England where more than 86% of pharmacists had Internet access at home, but only 43% had it at the workplace [12]. Nevertheless, despite their general confidence in the use of computers, the need for training on particular software and applications has been identified among pharmacists in England [12]. Moreover, with rapid advances in technology, online learning is now an integral part of undergraduate courses, complementing the traditional learning activities of healthcare professionals in many countries, including Europe, the USA, Australia, and Canada [13].

In Lebanon, there are gaps in computer literacy among university students upon admission to pharmacy education, as the level of schools varies in this regard, especially between public and private schools. Indeed, computer skills are not standardized and considered optional for schoolchildren; the official examination (baccalaureate) is still based on knowledge acquired in fields other than computer skills. Additionally, in the absence of a national pharmacy core competency framework, gaps also exist in computer information delivered through academic pharmacy programs in Lebanon. Some curricula require high levels of computer skills, while some others rely less on that aspect; some pharmacy schools use a learning management system, while others do not (general observation). Also, due to the absence of this framework, the use of a dispensing software in community pharmacies is not mandatory and is at pharmacists’ discretion. Consequently, community pharmacies that are still not computerized are allowed to welcome pharmacy students/trainees, similar to the ones that are computerized. Moreover, computer literacy is expected to vary by age and personal efforts of the pharmacists [14, 15].

Nevertheless, drawing inspiration from advancements and initiatives in other countries, the Order of Pharmacists of Lebanon (OPL, the official pharmacists’ association) believed in the importance of involving Lebanese pharmacists in e-services; for this purpose, in addition to the OPL mobile application, the OPL introduced new online platforms to improve the practice of pharmacy, such as the Lebanese advanced patient profile, medication safety reporting, e-prescription, and most importantly, a learning management system (LMS) for online courses [16–20]. The latter was acquired by the OPL to help pharmacists complete their required number of credits as per Law 190. This law does not impose any database or reference; it stipulates that all registered pharmacists living in Lebanon should complete 15 yearly continuing education (CE) credits (equivalent to 15 h), of which 5 at least should be face-to-face; all courses and activities organized by the OPL should be offered to pharmacists free of charge [21].

In this context, most pharmacists agree that CE programs positively affect their practice and increase their knowledge, with computer-based courses being their preferred type of CE (60.6%) [22]. However, the route to using medical e-services and e-learning is not straightforward. High setup costs and time commitments to sustain quality are issues brought up in this respect [15]. Therefore, the objectives of this study were to validate the general confidence with computer use (GCWCU) scale and assess computer literacy and its associated factors among Lebanese pharmacists.

## Methods

### Study design

This cross-sectional study was conducted between February and May 2018 among a sample of 628 Lebanese pharmacists. All pharmacists were eligible to participate: out of a total of 7974 registered pharmacists, 3762 (47.2%) were community practitioners, 803 (10.1%) medical representatives, 571 (7.2%) managers in pharmaceutical companies, 283 (3.5%) hospital pharmacists, and 169 (2.1%) academic pharmacists, while 2004 (25.1%) were retired or not working. The sample consisted of those who agreed to complete the questionnaire among those approached.

### Data collection

Data collection was performed by a team of study-independent pharmacists. The latter are full-time inspectors at the OPL and had been thoroughly trained by the research team prior to starting the study. After verbal agreement from participants, the OPL inspectors explained the study objectives stated at the beginning of the questionnaire before distributing it and gave no further information to avoid influencing respondents.

### Sample size calculation

In Lebanon, pharmacists cannot practice unless they are registered with the OPL. By October 2017, the total number of registered pharmacists was 7974. The Epi-info software was used to calculate the required sample size, taking a confidence level of 95%, a risk of error of 5%, and assuming that 50% of the pharmacists have good computer literacy in the absence of similar studies in the country. A minimal sample of 350 pharmacists was calculated. The questionnaire was distributed to a sample of 750 pharmacists to take refusals into account. Of the 750 targeted, 628 (83.73%) pharmacists filled out and returned the questionnaire.

### Survey instrument

The self-administered questionnaire was developed and reviewed by ten academics and pharmacy practitioners and required 15–20 min to complete. It was piloted on a sample of 10 pharmacists and did not require any modification, thus the data were included in the study. The version of the questionnaire used in this study is presented in Supplementary material 1. The questionnaire was available in both English and French, the 2 languages of pharmacy education in Lebanon. It was initially developed in English and translated into French by a pharmacist fluent in both languages. The French version was then translated back into English by another pharmacist, not aware of the English version. Both English and French versions were then compared and required no significant corrections. More details about the methodology used can be found in other studies [14, 15].

The questionnaire comprised three distinct sections. The first section clarified the socio-demographic characteristics, in addition to years of experience, the number of working hours per day, and the highest degree achieved. The second section focused on technology and included questions about the available connected devices at the pharmacy, the type of smartphone owned by the pharmacist, time spent over the Internet per day, and questions about the difficulty of accessing the OPL e-library and the LMS platform to take online courses. This section also included four questions on the perceived value of CE and nine on the motivation for undertaking CE [23]; both scales were inspired from a previous study, which included a theoretical framework for continuing education for professionals and its application among pharmacists [24] and validated in Lebanon [25].

In this section also, the “general confidence with computer use scale” (GCWCU) [26], a 12-item tool originally created in Australia, was used to assess computer literacy among Lebanese pharmacists. A thorough literature search showed that all the scales assessing computer literacy among pharmacists were specific to particular populations and activities and, therefore, could not be adapted to the Lebanese context [9, 27]. The GCWCU scale [26] used in this study was the only tool measuring computer literacy in the general population; it was thus adapted to assess computer literacy among Lebanese pharmacists. However, the last question in this scale, which initially assessed the ability to learn mathematics using computers, was replaced by a question on the ability to take CE courses using computers. Answers were scored on a 5-point Likert scale from 1 (strongly disagree) to 5 (strongly agree). The total score was calculated by adding the answers to all the questions, with higher scores indicating higher self-reported computer literacy.

The third section was designed to assess the pharmacists’ communication with the OPL and included questions on having the OPL mobile application, reading OPL messages (through the application or by short message text), being aware of the number of CE credits earned to date, and being aware of the total credits required by the end of December 2019 (end of the CE cycle).

### Statistical analysis

Statistical analyses were performed using the Statistical Package for Social Science (SPSS) version 23. Descriptive statistics were computed for continuous variables (mean ± standard deviation) and categorical variables (counts and percentages). To confirm the GCWCU scale construct validity in the Lebanese population, an exploratory factor analysis was launched for the 12 items of the questionnaire, using the principal component analysis technique; the extracted factors were correlated to each other, so a Promax rotation was applied. The Kaiser-Meyer-Olkin (KMO) measure of sampling adequacy and Bartlett’s test of sphericity were checked for the model’s adequacy. Factors with Eigenvalues higher than one were retained. The reliability of the scale and different subscale factors was assessed using Cronbach’s alpha.

In the bivariate analysis, the Student *t* test was used to compare the means of two groups, and the ANOVA test to compare the means of 3 groups or more. The Pearson correlation coefficient was used to correlate between quantitative variables. A stepwise multivariable linear regression model was conducted to examine factors associated with the self-reported GCWCU score. Variables that showed a *p* < 0.2 in the bivariate analysis were entered in the multivariable model to decrease confounding. In all cases, a *p* value of 0.05 was considered statistically significant.

## Results

### Sociodemographic characteristics

Of the 750 questionnaires distributed, 628 (83.37%) were completed and collected back. The sociodemographic characteristics of the participants are summarized in Table 1. The mean age of the participants was 39.04 ± 10.57 years, 66.9% were females, 41.1% had a bachelor degree in pharmacy and worked in Mount Lebanon, and 62.4% were community pharmacy owners. Also, for the whole sample, our results showed a mean of 13.32 years of practicing pharmacy (SD = 9.42), 6.01 working days per week (SD = 1.11), and 10.56 working hours per day (SD = 6.03).

**Table 1:** Characteristics of the sample population

		Frequency (%)
Gender	Male	208 (33.10%)
	Female	420 (66.90%)
Education level	BS Pharmacy	258 (41.10%)
	PharmD	249 (39.60%)
	Masters	98 (15.60%)
	PhD	23 (3.70%)
University you graduated from	LU	115 (18.30%)
	USJ	147 (23.40%)
	BAU	89 (14.20%)
	LAU	55 (8.80%)
	LIU	61 (9.70%)
	AUB	3 (0.50%)
	Outside Lebanon	157 (25.00%)
Work location	Not working	19 (3.00%)
	Beirut	92 (14.60%)
	Mount Lebanon	258 (41.10%)
	North Lebanon	20 (3.20%)
	South Lebanon	120 (19.10%)
	Bekaa	119 (18.90%)
Sector of work	Not working	19 (3.00%)
	Community employer	391 (62.40%)
	Community employee	122 (19.50%)
	Hospital/clinical	28 (4.50%)
	Scientific office/medical representative	37 (5.90%)
	Academia	11 (1.80%)
	Public sector (MOPH)	14 (2.20%)
	Industry	6 (1%)
**Computer- and technology-related characteristics**		
Owning a computer	No	255 (40.60%)
	Yes	373 (59.40%)
Owning a smart phone	No	273 (43.50%)
	Yes	355 (56.50%)
Owning a tablet	No	565 (90.00%)
	Yes	63 (10.00%)
Connected to the internet for at least 4 h per day	No	54 (8.60%)
	Yes	549 (87.40%)
	Only when needed	25 (4.00%)
Ease of accessing OPL e-library account	Never tried	70 (11.10%)
	Easy	242 (38.50%)
	Intermediate	211 (33.60%)
	Difficult	105 (16.70%)
Ease of accessing OPL Swank platform	Never tried	94 (15.00%)
	Easy	205 (32.60%)
	Intermediate	216 (34.40%)
	Difficult	113 (18.00%)
Having OPL mobile application	No	96 (15.30%)
	Yes	532 (84.70%)
Read OPL messages through the application or by text message	No	27 (4.30%)
	Yes	515 (820%)
	Sometimes	86 (13.70%)
Know the number of CE credits earned to date	No	294 (46.80%)
	Yes	216 (34.40%)
	More or less	118 (18.80%)
Know the number of CE credits to be achieved by December 2019	No	311 (49.50%)
	Yes	198 (31.50%)
	More or less	119 (18.90%)

*LU* Lebanese University, *USJ* Saint-Joseph University, *BAU* Beirut Arab University, *LAU* Lebanese American University, *LIU* Lebanese International University, *AUB* American University of Beirut

### Factor analysis

The factor analysis for this scale was run over the whole sample (total *n* = 628). The scale items converged over a solution of two factors that had an Eigenvalue over 1, explaining a total of 57.10% of the variance. All items could be extracted from the list, since no items over-correlated to each other (*r* > 0.9), had a low loading on factors (< 0.3), or a low communality (< 0.3). A Kaiser-Meyer-Olkin measure of sampling adequacy of 0.88 was found, with a significant Bartlett’s test of sphericity (*p* < 0.001). Table 2 summarizes the components obtained according to the Promax rotated matrix. Moreover, the Cronbach’s alpha for the whole scale was 0.72.

**Table 2:** Promax rotated matrix of the general confidence with computer use scale

Factor	Items	Factor 1	Factor 2
I have never felt myself able to learn how to use computers.	5	0.87	
It takes me much longer to understand how to use computers than the average person.	4	0.84	
I find having to use computers frightening.	7	0.84	
I find using computers confusing.	11	0.80	
I’m nervous that I’m not good enough with computers to be able to use them for continuing education.	12	0.71	
I have never been very excited about using computers.	10	0.66	
I am not what I would call a computer person.	3	0.64	
I don’t understand how some people can enjoy spending so much time using computers.	9	0.63	
I find it easier to learn how to use a computer than I do learning other things.	1		0.76
I enjoy trying new things on a computer.	6		0.75
When I have difficulties using a computer I know I can handle them.	2		0.72
I find many aspects of using computers interesting and challenging.	8		0.71
**Cronbach’s alpha**		0.79	0.73
**Percentage of variances explained**		41.45	15.65

Cronbach alpha for the total scale = 0.716

Kaiser-Meyer-Olkin measure of sampling adequacy = 0.878; Bartlett’s test of sphericity (*p* < 0.001)

### Descriptive and bivariate analysis related to the GCWCU

The mean self-reported GCWCU score was 43.40 ± 7.31 (median 44), with a minimum of 23 and a maximum of 60. In the absence of a cutoff value for this scale, the median was used as a cutoff point. The results showed that 316 (50.30%) of pharmacists had good computer literacy (score ≥ 44).

The bivariate analysis of factors associated with the GCWCU score showed that lower scores were significantly associated with higher age (*r* = − 0.26; *p* < 0.001), a higher number of years of practicing pharmacy (*r* = − 0.23; *p* < 0.001), and higher working days per week (*r* = − 0.10; *p* = 0.015). On the other hand, a higher mean GCWCU score was found in pharmacists with a PhD degree (47.08), graduated from the Lebanese American University (45.78), working in Beirut (44.98), working in academia (51.09), connected to the internet for at least 4 h per day (43.86), and in those who find it easy to access the OPL e-library (46.05) and the OPL LMS platform (46.06). Also, a higher mean GCWCU score was found in pharmacists who had a computer (44.09 vs 42.39), a smartphone (44.16 vs 42.41), or a tablet (47.17 vs 42.98) and in those who had mobiles with iOS operating system (44.61 vs 42.95) compared to those who did not (Table 3).

**Table 3:** Bivariate analysis of factors associated with the general confidence with computer use scale

Variable	GCWCU scale	***p***
**Gender**		0.854
Male	43.48 ± 7.30	
Female	43.36 ± 7.32	
**Educational level**		**0.001**
BS Pharmacy	42.15 ± 6.98	
PharmD	44.17 ± 7.62	
Master degree	43.86 ± 6.62	
PhD	47.08 ± 8.12	
**University you graduated from**		**< 0.001**
Lebanese University	44.74 ± 6.86	
Saint-Joseph University	43.95 ± 7.51	
Beirut Arab University	43.06 ± 7.32	
Lebanese American University	45.78 ± 7.83	
Lebanese International University	44.77 ± 6.79	
American University of Beirut	45.00 ± 3.60	
Outside Lebanon	40.73 ± 6.80	
**Sector of work**		**0.001**
Not working yet	44.50 ± 7.47	
Community employer	42.40 ± 7.16	
Community employee	43.93 ± 7.03	
Hospital/clinical	46.64 ± 7.16	
Scientific office/medical representative	44.45 ± 6.21	
Academia	51.09 ± 7.87	
Public sector (MOPH)	47.71 ± 7.22	
Industry	48.83 ± 11.05	
**Connected to the internet for at least 4 h per day**		**< 0.001**
No	39.75 ± 6.26	
Yes	43.86 ± 7.32	
As needed	41.12 ± 6.64	
**Ease to access OPL e-library account**		**< 0.001**
Never tried	41.08 ± 7.35	
Easy	46.05 ± 6.50	
Intermediate	41.96 ± 7.03	
Difficult	41.73 ± 7.83	
**Ease to access OPL Swank platform**		**< 0.001**
Never tried	42.35 ± 7.79	
Easy	46.06 ± 6.50	
Intermediate	42.71 ± 7.08	
Difficult	40.76 ± 7.31	
**Computer ownership**		**0.004**
No	42.39 ± 7.05	
Yes	44.09 ± 7.41	
**Smartphone ownership**		
No	42.41 ± 6.98	**0.003**
Yes	44.16 ± 7.47	
**Tablet ownership**		**< 0.001**
No	42.98 ± 7.18	
Yes	47.17 ± 7.40	
**iOS phone type**		**0.012**
No	42.95 ± 7.24	
Yes	44.61 ± 7.38	
**Android phone type**		0.443
No	43.59 ± 7.19	
Yes	43.14 ± 7.48	

Numbers in bold indicate significant *p*-values

### Multivariable analysis

The multivariable analysis, taking the self-reported GCWCU score as the dependent variable, showed that a higher GCWCU score was significantly associated with pharmacists who find the access to the e-library easy compared to those who never tried (beta = 2.58), have a tablet compared to those who have not (beta = 2.80), are connected for at least 4 h per day compared to not (beta = 2.72), find it easy to access the OPL LMS platform compared to those who never tried (beta = 2.39), work in a hospital compared to those not working (beta = 2.60), and hold a PhD (beta = 4.28) or a PharmD degree (beta = 1.16) compared to those with a bachelor degree. On the other hand, a lower GCWCU score was significantly associated with higher age (beta = − 0.19) and working in South Lebanon compared to not working (beta = − 1.76) (Table 4).

**Table 4:** Multivariable analysis: stepwise linear regression taking the general confidence with computer use scale score as the dependent variable

Variable	Unstandardized beta	Standardized beta	***p*** value	95% confidence interval	
Finding e-library access easy compared to never tried	2.58	0.17	0.001	1.11	4.05
Age	– 0.19	– 0.28	< 0.001	– 0.24	– 0.14
Owning a tablet	2.80	0.12	0.001	1.09	4.51
South Lebanon compared to those not working	– 1.76	– 0.10	0.01	– 3.10	– 0.43
Connected at least 4 h per day	2.72	0.12	0.001	1.16	4.27
Finding OPL Swank access easy compared to never tried	2.40	0.15	0.002	0.86	3.93
PhD degree compared to bachelor degree	4.29	0.11	0.002	1.52	7.06
PharmD degree compared to bachelor degree	1.17	0.08	0.034	0.09	2.25
Hospital pharmacists compared to non-working	2.60	0.07	0.04	0.12	5.09

## Discussion

This study validated the GCWCU scale for use among pharmacists in Lebanon. The factor analysis of the GCWCU scale showed that all items converged over two factors, contrary to the original scale [26] that converged over only one factor. This might be due to cultural differences between Australia and Lebanon. The internal consistency of the GCWCU scale in this study was lower than that of the original [26], with Cronbach’s alpha values of 0.716 and 0.92, respectively. Nevertheless, our findings show that the scale is satisfactory overall and can be used to assess computer literacy among Lebanese pharmacists.

Our results also showed that 50.3% of the Lebanese pharmacists self-reported being computer literate; although this percentage is higher than that of Italian pharmacists [28], it is still considered low. A previous study conducted in 2006 had highlighted that most pharmacy schools had not implemented computer learning courses in pharmacy, which may possibly explain the lack of suitable expertise in academia [29]. Pharmacy schools are expected to conduct computer courses (i.e., how to sue a computer) and teach students about the use of learning materials that are provided via learning management systems. Consequently, it is necessary to take educational and professional measures to provide appropriate learning, thus promoting computer skills among pharmacists, whether during undergraduate studies or later in the context of continuing education; hence, the need to include relevant courses in the pharmacy curriculum of all Lebanese universities seems warranted.

The use of computers is affected by many socio-demographic factors, such as age and education level [30]. Increased age was associated with lower computer literacy among pharmacists. Younger people, who grew up with computers, are believed to be more computer literate than older people who became familiar with this new technology at a later age. Conversely, results from previous research have shown that older adults have significantly improved many facets of their computer literacy over the past five years compared to university students [31].

Our results showed that higher self-reported GCWCU scores were significantly associated with pharmacists who find it easy to access the OPL e-library compared to those who never tried, have a tablet compared to those who have not, are connected for at least 4 h per day compared to not, find it easy to access the OPL LMS compared to those who never tried, hold a PhD or a PharmD degree compared to those with a bachelor degree, and work in a hospital compared to those who do not work at all. These results confirm those of previous studies showing that education and years of experience are the most important predictors of the knowledge amount and strategic internet skills [32–34] and that some faculty members (PhD holders) in pharmacy schools might help students acquire essential computer skills [35].

Hospital pharmacists had higher confidence with computer use than non-working pharmacists. This was also expected since computer management (the use of computers and computerized records) in hospitals is an integral part of the pharmacist’s duties, with hospital pharmacists feeling comfortable using such resources [9]. In fact, a previous study has shown that the majority (60%) of Lebanese hospital pharmacists preferred computer/Internet-based CE programs [22]. This can be explained by the fact that hospital pharmacists are often involved in therapy management programs, medication incompatibilities, and electronic prescriptions, all of which require a greater use of technology and up-to-date information [12]. For example, in the USA, a survey conducted in 2013 on 465 hospital pharmacy departments showed that almost all hospital healthcare services are based on electronic health records. Hence, computer use is increasingly becoming a sign of value and quality in these institutions [29].

### Limitations

This study has some limitations. Its cross-sectional design precludes causality assessment between dependent and independent variables. Selection biases can arise from the refusal rate and the small sample size that may not be representative of the general population. Since community employers were the most represented in our sample and some categories (e.g., hospital pharmacists) underrepresented, the study results cannot be extrapolated. Moreover, several information biases could be noted: under- or overestimation of associations due to social desirability (the tendency of survey respondents to answer questions in a manner that will be viewed favorably by others), recall bias, and misinterpretation of the questions. Furthermore, a confirmatory factor analysis and reliability results (test-retest, interrater and intra-rater validity) were not conducted in the validation part. Larger-scale studies, taking into account these potential biases, are suggested.

## Conclusion

This study showed that the GCWCU is adequate to assess computer literacy in Lebanese pharmacists and identified factors affecting and/or associated with computer literacy. It presented insights into essential computer skills and abilities of Lebanese pharmacists and identified factors associated with their general confidence with computer use in their practice. These findings would help decision-makers and CE providers design learning materials for pharmacists to improve their computer literacy for better practice and patient care.

## Data Availability

The authors do not have the right to share any data information as per their institution’s policies.

## Abbreviations

OPL: Order of Pharmacists of Lebanon
LMS: Learning management system
CE: Continuing education
GCWCU: General confidence with computer use scale
KMO: Kaiser-Meyer-Olkin
